# On the Performance of Interleavers for Quantum Turbo Codes

**DOI:** 10.3390/e21070633

**Published:** 2019-06-27

**Authors:** Josu Etxezarreta Martinez, Pedro M. Crespo, Javier Garcia-Frías

**Affiliations:** 1 Department of Basic Science, Tecnun-University of Navarra, 20018 San Sebastian, Spain; 2 Department of Electrical and Computer Engineering, University of Delaware, Newark, DE 19716, USA

**Keywords:** quantum error correction, turbo codes, interleavers, error floors

## Abstract

Quantum turbo codes (QTC) have shown excellent error correction capabilities in the setting of quantum communication, achieving a performance less than 1 dB away from their corresponding hashing bounds. Existing QTCs have been constructed using uniform random interleavers. However, interleaver design plays an important role in the optimization of classical turbo codes. Consequently, inspired by the widely used classical-to-quantum isomorphism, this paper studies the integration of classical interleaving design methods into the paradigm of quantum turbo coding. Simulations results demonstrate that error floors in QTCs can be lowered significantly, while decreasing memory consumption, by proper interleaving design without increasing the overall decoding complexity of the system.

## 1. Introduction

Classical turbo codes, introduced by Berrou et al. in Reference [1], marked a breakthrough in classical coding theory as the first practical codes closely approaching Shannon’s channel capacity [2]. Motivated by their success, serial concatenation of Quantum Convolutional Codes (QCC) was proposed in Reference [3]. The use of a serial structure in Reference [3] is explained by the laws of quantum mechanics, in particular by the underlying classical-to-quantum isomorphism [4] applied in the design of quantum error correction codes (QECC): Classical turbo codes are generally systematic, interleaved parallel concatenations of convolutional codes but in the quantum domain such a systematic parallel structure is prohibited by the no-cloning theorem.

The performance of the Quantum Turbo Codes (QTCs) proposed in Reference [3] is not on par with that of classical serial turbo codes. The reason is that unassisted QCCs cannot be both non-catastrophic and recursive at the same time, properties that are required for the inner code of a turbo code so that the minimum distance grows with the block length and the iterative decoding algorithm achieves convergence. Consequently, non-catastrophic, non-recursive QCCs were used in Reference [3], resulting in codes with bounded minimum distance. Utilizing the entanglement-assistance (EA) techniques (Entanglement-assistance techniques utilize entangled qubits pre-shared between the sender and the receiver, which simplifies the construction of stabilizer QECCs by relaxing the stringent commutativity conditions that the stabilizers need to satisfy.) for QECCs presented in Reference [5], this issue was addressed by Wilde et al. in [6], which proposed QTCs operating within 1 dB of their corresponding EA hashing bounds. Taking a step further, Babar et al. proposed in Reference [7] an EXtrinsic Information Transfer (EXIT) chart technique to narrow the gap to the Hashing bound, resulting in codes with a performance as close as 0.3 dB to the hashing bounds. However, these QTC codes present error floors that are higher than in the original distance-spectra-based codes in Reference [6]. Based on the use of Quantum IrRegular Convolutional Codes (QIRCC) as outer codes, the authors in Reference [8] showed a performance in the turbo-cliff region similar to that of the EXIT optimized codes in Reference [7] but with lower error floors. An entanglement-assisted version of QIRCCs was introduced in Reference [9] with the aim of designing efficient concatenated QECCs for asymmetric depolarizing channels. Finally, a Quantum Unity Rate Code (QURC) aided QTC scheme was proposed in Reference [10] in order to improve the performance of the outer code without experiencing code rate reduction due to the inner code.

The serial concatenation of the inner and outer QCCs used to construct QTCs is realized by means of an interleaver, which permutes the symbols so that the error locations are randomized and error correction can be improved. The reason for the use of an interleaver in concatenated coding schemes is that the first stage in the decoding process generates bursts of errors that are more efficiently corrected in the second stage if they are scrambled. The QTCs proposed in the literature [3,4,5,6,7,8,9] use the so-called random interleaver. However, it is known from classical turbo codes that interleaving design plays a central role in optimizing performance, specially when the error floor region is considered [11,12,13,14,15,16,17,18]. Motivated by such studies, in this paper we investigate the application of different types of interleavers in QTCs, aiming at reducing the error floors. Simulation results show that the QTCs designed using the proposed interleavers present the same behavior in the turbo-cliff region as the codes with random interleavers in Reference [7], while the performance in the error floor region is improved by up to two orders of magnitude. Simulations also show reduction in memory consumption, while the performance is comparable to or better than that of QTCs with random interleavers.

The remainder of this paper is organized as follows: Section 2 presents the QTC system model and presents the classical interleavers considered in the quantum codes; Section 3 presents Monte Carlo simulations showing that interleaver construction is beneficial to lower the error floor of the considered QTCs; finally, Section 4 provides the conclusions reached in this paper.

## 2. Classical Interleavers for Quantum Turbo Codes

### 2.1. System Model

**Figure 1:**
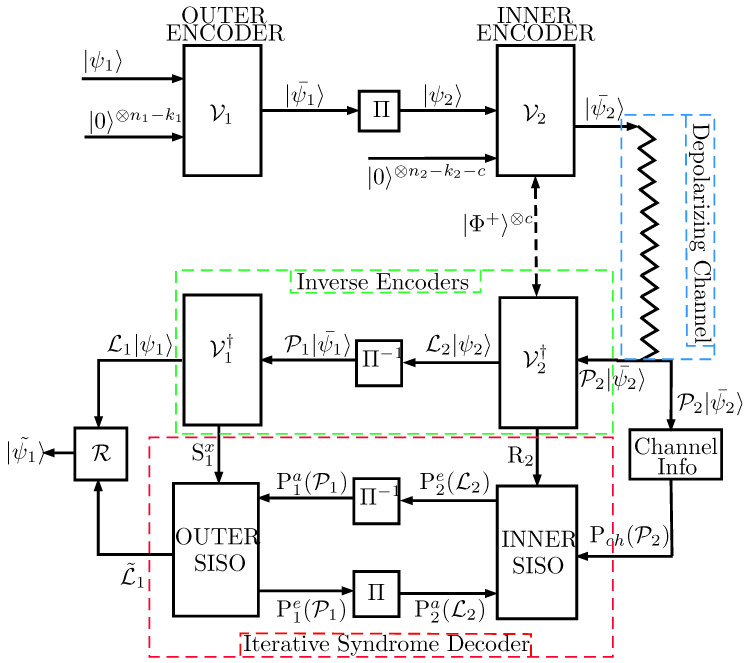
Schematic of the Quantum Turbo Code (QTC). Note that the *c* pre-shared EPR pairs ${|\Phi \rangle }^{+}$ are needed for the inner encoder to be both recursive and non-catastrophic [6]. $P_{i}^{a}\left(.\right)$ and $P_{i}^{e}\left(.\right)$ denote the a-priori and extrinsic probabilities related to each of the SISO decoders used for turbo decoding.

The Quantum Turbo Codes considered in this paper consist of the interleaved serial concatenation of unassisted QCCs acting as outer codes and entanglement-assisted QCCs inner codes. Figure 1 presents the full schematic representation of such quantum error correction system. The *k* input logical qubits that compose the information word $|\psi_{1}\rangle$ are first fed to the outer $\left[n_{1},k_{1},m_{1}\right]$ unassisted convolutional encoder $V_{1}$ and encoded into $n^{\prime }=k\frac{n_{1}}{k_{1}}$ physical qubits with the help of $\left(n_{1}-k_{1}\right)$ ancilla qubits and *m* memory qubits. The $n^{\prime }$ physical qubits that form the codeword $|\bar{\psi_{1}}\rangle$ generated by the first encoder are then passed through a quantum interleaver Π before being input to the inner convolutional encoder $V_{2}$. Such an encoder is an $\left[n_{2},k_{2},m_{2},c\right]$ entanglement-assisted encoder that encodes the interleaved sequence of $n^{\prime }$ qubits $|\psi_{2}\rangle$ into the codeword $|\bar{\psi_{2}}\rangle$ of length $n=n^{\prime }\frac{n_{2}}{k_{2}}=k\frac{n_{2}}{k_{2}}\frac{n_{1}}{k_{1}}$, aided by $\left(n_{2}-k_{2}-c\right)$ ancilla qubits, *c* pre-shared EPR pairs and $m_{2}$ memory qubits. Codeword $|\bar{\psi_{2}}\rangle$ is then transmitted through a quantum depolarizing channel (The depolarizing channel is a widely used channel model in order to represent the decoherence effects that produce errors in quantum information [3,4,5,6,7].) with depolarizing probability *p* inflicting an *n*-qubit Pauli error $P_{2}\in G_{n}$ to the codeword. The operation of the depolarizing channel on an individual qubit with density matrix ρ is defined as

(1) $$N_{D}\left(\rho \right)=\left(1-p\right)\rho +\frac{p}{3}\left(X\rho X+Y\rho Y+Z\rho Z\right),$$

where *p* is the depolarizing probability and X,Y,Z are the Pauli matrices. The depolarizing channel is independently applied to each of the qubits of the stream $|\bar{\psi_{2}}\rangle$, and, consequently, each of the qubits experiences a bit-flip (X operator) with probability p/3, a phase-flip (Z operator) with probability p/3 or a combination of both (Y operator) with probability p/3.

At the output of the depolarizing channel, the state $P_{2}|\bar{\psi_{2}}\rangle$ is fed to the inverse of the inner encoder $V_{2}^{†}$, which outputs the decoded state $L_{2}|\psi_{2}\rangle$, where $L_{2}\in G_{n^{\prime }}$ refers to the logical error suffered by the decoded state due to the operation of the channel; and the classical syndrome bits $R_{2}=\left(S_{2}^{x},E_{2}^{x,z}\right)$ obtained from *Z* basis measurements on the ancilla qubits and Bell measurements on the pre-shared EPR pairs. The corrupted logical qubits are then passed through a de-interleaver $\Pi^{-1}$ resulting in the state $P_{1}|\bar{\psi_{1}}\rangle$, which is supplied to the inverse of the outer encoder $V_{1}^{†}$. The resulting output is the state $L_{1}|\psi_{1}\rangle$, which corresponds to the information quantum state corrupted by a logical error $L_{1}\in G_{k}$; and the classical syndrome bits $S_{1}^{x}$ obtained after measuring the ancilla qubits on the *Z* basis. The classical syndromes $R_{2}$ and $S_{1}^{x}$, obtained in the inverse decoders $V_{2}^{†}$ and $V_{1}^{†}$, respectively, are then provided to the iterative syndrome decoder consisting of two serially concatenated Soft-In Soft-out (SISO) decoders, as shown in Figure 1. Based on $R_{2}$ and $S_{1}^{x}$, as well as the channel information $P_{ch}\left(P_{2}\right)$, both SISO decoders engage in degenerate iterative decoding [3,6] to estimate the most likely error coset ${\tilde{L}}_{1}$ that has corrupted the information quantum state. Based on such an estimation, a recovery operation R is applied to the corrupted state $L_{1}|\psi_{1}\rangle$, yielding the recovered output $|\tilde{\psi_{1}}\rangle$.

As already mentioned, the aim of this paper is to show that interleaver design is beneficial to reduce the error floors of QTCs. To that end, the inner and outer QCCs used to construct the concatenated error correcting system presented in Figure 1 are the EXIT-optimized codes introduced in Reference [7]. The parameters of the inner and outer codes are presented in Table 1. Such configuration, utilizing a random interleaver for the serial concatenation of the constituent QCCs, was studied extensively in Reference [7]. Therefore, it is an excellent benchmark for the interleaver constructions proposed in this paper.

From the configuration parameters presented in Table 1, it can be seen that the QTCs used in this paper will be rate 1/9 turbo codes with an entanglement consumption rate of 6/9. The noise limit $p^{*}$ can be found from the entanglement-assisted hashing bound, which for such parameters is $p^{*}=0.3779$ [6].

### 2.2. Classical Interleavers for QTCs

The concatenation between the outer and inner QCCs is done by the quantum interleaver Π of size *N*, which is an *N* to *N*-qubit symplectic transformation (The notation we utilize to describe the operation of a quantum interleaver is the so-called symplectic notation as used in Reference [3].) composed by a permutation π of the *N* qubit registers and a tensor product of single-qubit symplectic transformations. It acts by multiplication on the right on $G_{N}$ as

(2) $$\left(P_{1},\cdots ,P_{N}\right)\to \left(P_{\pi (1)}K_{1},\cdots ,P_{\pi (N)}K_{N}\right),$$

where $K_{1},\cdots ,K_{N}$ are some fixed symplectic matrices acting on $G_{1}$, where $G_{N}$ refers to the symplectic representation of the *N*-fold Pauli group [3].

In order to use classical interleavers in QTCs, they must properly match the definition of quantum interleavers in (2). Classical interleavers consist only of permutations of the information arrays with no such thing as individual symplectic transformations. Consequently, those operations will be taken as identity operators acting on the registers $K_{i}=I,\forall i$; and so the only non-trivial operation of such quantum interleavers will be the scrambling of the qubits in the quantum information stream (The random interleavers used in References [3,4,5,6,7,8,9] also follow this approach.). Following this approach, the quantum interleavers presented in this paper will be *N* to *N* qubit symplectic transformations acting as

(3) $$\left(P_{1},\cdots ,P_{N}\right)\to \left(P_{\pi (1)},\cdots ,P_{\pi (N)}\right)$$

to the qubit data stream, and where the permutation pattern π corresponds to the classical interleaver under consideration.

### 2.3. Considered Classical Interleavers

All the QTC schemes in References [3,4,5,6,7,8,9] are based on *random interleavers*, that is, the interleaving patterns π are selected at random. However, it is known from classical turbo codes that the usage of interleavers with some added structure is beneficial to reduce either the error floors or the memory requirements [11,12,13,14,15,16,17,18]. In order to show that interleaver design is also beneficial when implementing quantum turbo codes, we consider the following three types of classical interleavers.

The first classical interleavers considered here are the *S-random* interleavers [11]. They are randomly generated by imposing the following condition on the interleaving distance or spread (*S*):

(4) |π(i)−π(j)|>Sforiandjwith|i−j|≤S.

If the heuristic recommendation $S<\sqrt{\frac{N}{2}}$ is satisfied, where *N* is the blocklength, *S*-random interleavers can usually be produced in reasonable time by repeatedly generating random integers until condition (4) is satisfied [11,15]. However, as indicated in Reference [18], reasonable values of *S* are sometimes lower when *N* is large. We next consider the deterministic *Welch-Costas* [17] interleaver, which is defined by two parameters: p=N+1, a prime number, and α, a primitive element modulo *p*. Once α is chosen for the selected blocklength, it is proven in Reference [17] that

(5) $$\pi \left(i\right)=\left(\alpha^{i}\;\mathrm{mod}\;\left(N+1\right)\right)-1,\;\forall i\in \left\{0,\cdots ,N-1\right\}$$

forms a permutation which is selected as the interleaving rule. Although the spreads obtained with Welch-Costas interleavers are low, their dispersion parameter, γ, is maximized. The dispersion of an interleaver π is a parameter that represents the randomness of the permutation, and it is calculated as [17]

(6) γ=|D(π)|N2,

where |·| indicates cardinality and D(π) is the set of *displacement vectors* of π defined as

(7) D(π)={(j−i,π(j)−π(i))|0≤i<j<N}.

The final classical interleaver we will considered for quantum error correction is the *JPL* interleaver [17], recommended in the “CCSDS Recommendation for Telemetry Channel Coding” standard [19] because it has good spreading and dispersion parameters. The permutations that define this family of interleavers are constructed by applying the following algorithm:

- Factorize the length of the interleaver $N=k_{1}k_{2}$, where $k_{1}=8$ usually.
- For s=1 to s=N do
  1. m=(s−1)mod2
  2. $i=\lfloor \frac{s-1}{2k_{2}}\rfloor$
  3. $j=\left\lfloor \frac{s-1}{2}\right\rfloor -ik_{2}$
  4. $t=\left(19i+1\right)\;\mathrm{mod}\;\frac{k_{1}}{2}$
  5. q=tmod8+1
  6. $c=\left(p_{q}j+21m\right)\;\mathrm{mod}\;k_{2}$
  7. $\pi \left(s\right)=2\left(t+c\frac{k_{1}}{2}+1\right)-m$

where $p_{q}$ is defined as the primes $p_{1}=31,p_{2}=37,p_{3}=43,p_{4}=47,p_{5}=53,p_{6}=59,p_{7}=61,p_{8}=67$.

## 3. Results and Discussions

In this section we present Monte Carlo simulations to asses the performance of quantum turbo codes when using the interleavers proposed in Section 2.3. To that end, the EXIT chart optimized QCCs defined by the parameters in Table 1 have been used. The interleaver length is N=3000, so that the input quantum information consists of 1000 qubits. As in Reference [7], a maximum of 15 iterations has been set for the turbo decoder.

In order to perform the numerical simulations, as done in References [6,7], an *n*-qubit error is randomly generated in each transmission round as explained in (1). At the decoder, the syndromes $R_{2}$ and $S_{1}^{x}$ are computed first, and the turbo decoding algorithm runs until the hard decisions on the estimated logical errors are the same as in the previous iteration or until the number of iterations reaches the maximum value (which we fix to 15).

The operational figure of merit selected in order to evaluate the performance of these quantum error correction schemes is the *Word Error Rate* (WER), which is the probability that at least one qubit of the received block is incorrectly decoded.

Regarding the numerical Monte Carlo methods used in order to estimate the WER of the different QTCs, the following rule of thumb has been used [20] in order to select the number of blocks to be transmitted, $N_{blocks}$:

(8) $$N_{blocks}=\frac{100}{WER}.$$

As explained in Reference [20], and under the assumption that the observed error events are independent, this results in a 95% confidence interval of about $\left(0.8\hat{WER},1.25\hat{WER}\right)$, where $\hat{WER}$ refers to the empirically estimated value for the WER.

The following interleavers have been implemented:

1. *S*-random interleaver with parameter S=25,
2. Welch-Costas interleaver with parameter α=2987 and
3. JPL interleaver.

The spread (*S*) and dispersion (γ) parameters of such designed interleavers are indicated in Table 2.

**Figure 2:**
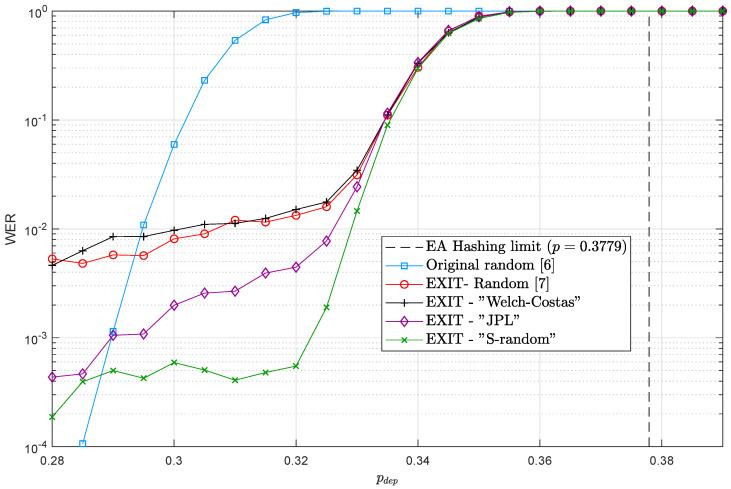
Word error rate (WER) performance curves for the 1/9-QTCs in Table 1 when different interleaving methods are used.

Figure 2 shows the results of Monte Carlo simulations for the different QTC schemes in the depolarizing channel. It can be seen that the performance of all QTCs is similar in the turbo-cliff region, achieving a gap (Note that the gap is calculated in a similar fashion as in Reference [7], that is, the distance between the QTC convergence threshold p=0.35 and the EA Hashing limit is taken as $10\log_{10}\left(0.3779/0.35\right)=0.3$ dB.) to the EA Hashing limit of 0.3 dB, which is also the gap obtained by the random interleaver QTCs of Reference [7]. However, different behaviors can be observed in the error floor region. As depicted in Figure 2, both the JPL and the *S*-random interleavers present a much lower error floor than that of random interleaving. Specifically, the error floor of the original ramdomly interleaved QTCs is of the order of $10^{-2}$, while the error floor of the *S*-random interleaver is around $10^{-4}$ and that of the JPL is of order of $10^{-3}$–$10^{-4}$. Notice that the error floor of the JPL interleaver presents a larger slope than that of the other interleavers, achieving levels close to those of the *S*-random interleaver when the channel quality improves. This means that quantum error correction systems using *S*-random and JPL interleavers present error floors around two orders of magnitude better than those obtained with random interleavers.

Figure 2 shows that the performance of the Welch-Costas interleaver is similar to that of the baseline random interleaver. However, the benefit of using the Welch-Costas interleaver is that its memory requirements are much smaller, as the permutation is defined just by parameters *N* and α. On the other hand, the random (and *S*-random) permutations must be stored completely. This means that using the Welch-Costas interleaver is a good option for systems with strict storage requirements. The memory requirements for the JPL interleaver are also lower than for the random and *S*-random ones, as the permutation can be defined by parameters *N*, $k_{1}$ and the primes $p_{q}$, while its performance is better than that of random interleavers but worse than that of *S*-random ones. Welch-Costas interleavers do have less memory requirements than JPL interleavers, but their performance is far worse in the error floor region. Table 3 summarizes the memory requirements for each of the interleavers. Note that both random and *S*-random interleavers require a memory consumption that increases linearly with *N*, while the memory requirements for the JPL and Welch-Costas interleavers are constant. Regarding the overall complexity of the decoding algorithm we have corroborated that it is practically the same for all interleavers.

As explained in Reference [17], the spread parameter is related to the number of distinct low weight error patterns that are possible to appear in the decoding, while the dispersion is related to the multiplicities of those existing low weight errors. Consequently, high spread is desired to avoid those harmful error sequences, while a dispersion parameter that is close to that of a random interleaver (γ≈0.81) will mean that those harmful errors will happen less regularly. This is why in classical turbo coding it is known that to achieve good error correction performance in the error floor region, an interleaver must have a high spread parameter and a dispersion parameter similar to the dispersion of random permutations [17]. The results presented in Figure 2 show that such rationale also applies to the QTC scenario.

## 4. Conclusions

We have adapted three interleaving design methods used for classical turbo codes to the quantum domain, demonstrating the importance of interleaver construction in quantum turbo codes. By using Monte Carlo simulations, we have shown that these constructions do enhance the error correction capability of quantum codes in the error floor region, leading to error rate improvements of up to two orders of magnitude. We have also shown that memory requirements can be lowered by using specific interleaver designs, while the performance in the error floor region is comparable or even better than that of the original EXIT optimized QTCs.

## Figures and Tables

**Table 1:** Parameters of the Quantum Convolutional Codes (QCC) encoders. *R* and *E* refer to the coding rate and the entanglement consumption rate, respectively. *m* refers to the memory qubits. The seed transformations U are represented using the decimal representation presented in Reference [6].

Config.	Encoder	R	E	m	Seed Transformation U
EXIT-optimized	Outer	1/3	0	3	${\left\{1048,3872,3485,2054,983,3164,3145,1824,987,3282,2505,1984\right\}}_{10}$
	Inner	1/3	2/3	3	${\left\{4091,3736,2097,1336,1601,279,3093,502,1792,3020,226,1100\right\}}_{10}$

**Table 2:** Spread and dispersion parameters.

Name	Spread (S)	Dispersion (γ)
Random [17]	1	≈0.81
*S*-random	25	0.8136
Welch-Costas	1	1
JPL	16	0.35

**Table 3:** Memory requirements.

Name	Parameters	Storage Requirements
Random [17]	π	*N*
*S*-random	π	*N*
Welch-Costas	*N*, α	2
JPL	*N*, $k_{1}$, $p_{q}$	10

## Abbreviations

The following abbreviations are used in this manuscript:

QECC	Quantum Error Correction Code
QCC	Quantum Convolutional Code
QTC	Quantum Turbo Code
QIRCC	Quntum IrRegular Convolutional Code
QURC	Quantum Unity Rate Code
EXIT	EXtrinsic Information Transfer
EPR	Einstein-Podoslky-Rosen
SISO	Soft-Input Soft-Output
JPL	Jet Propulsion Laboratory
CCSDS	Consultative Committee for Space Data Systems
WER	Word Error Rate
EA	Entanglement-Assisted
